# Time to Pay Attention? Information Search Explains Amplified Framing Effects Under Time Pressure

**DOI:** 10.1177/09567976211026983

**Published:** 2021-12-03

**Authors:** Ian D. Roberts, Yi Yang Teoh, Cendri A. Hutcherson

**Affiliations:** 1 Department of Psychology, University of Toronto Scarborough; 2 Department of Psychology, University of Toronto; 3 Rotman School of Management, University of Toronto

**Keywords:** attention, decision making, heuristics, risk taking, framing effect, dual systems, time pressure, adaptive, eye tracking, open data, open materials, preregistered

## Abstract

Decades of research have established the ubiquity and importance of choice biases, such as the framing effect, yet why these seemingly irrational behaviors occur remains unknown. A prominent dual-system account maintains that alternate framings bias choices because of the unchecked influence of quick, affective processes, and findings that time pressure increases the framing effect have provided compelling support. Here, we present a novel alternative account of magnified framing biases under time pressure that emphasizes shifts in early visual attention and strategic adaptations in the decision-making process. In a preregistered direct replication (*N* = 40 adult undergraduates), we found that time constraints produced strong shifts in visual attention toward reward-predictive cues that, when combined with truncated information search, amplified the framing effect. Our results suggest that an attention-guided, strategic information-sampling process may be sufficient to explain prior results and raise challenges for using time pressure to support some dual-system accounts.

Since the time of Plato, people have conceptualized their behavior as a struggle between capricious emotion and steadfast rationality. Modern researchers have often channeled this broad perspective when developing *dual-system models* that provide an intuitively appealing explanation for when and why people demonstrate systematic choice biases or behave in a more normative fashion (e.g., Kahneman, 2011; Kahneman & Frederick, 2002; Stanovich & West, 2000; but cf. De Neys, 2018; Evans, 2012; Pennycook et al., 2015). Particularly compelling evidence of a possible conflict between emotion and reason in choice biases has come from *framing effects*, in which superficial differences in the way a choice problem is described produce dramatic and seemingly irrational changes in preference. In this view, framing biases arise because of the operation of a fast, affective, and automatic process (System 1) and are mitigated with effort and time by slower, more “coolly” rational deliberation (System 2; Kahneman & Frederick, 2007). Although there have been mounting challenges to the theoretical (e.g., Grayot, 2020; Keren & Schul, 2009) and explanatory (e.g., Berkman et al., 2017; Hutcherson et al., 2015; Krajbich et al., 2015) utility of this perspective, some of the most compelling support for the dual-system account of framing effects continues to come from studies showing that they are amplified when choice time is limited (Diederich et al., 2018, 2020; Guo et al., 2017). It is therefore noteworthy that recent work suggests that time-pressure-induced changes in preferences previously attributed to dual systems can sometimes emerge from the boundedly rational, prioritized deployment of visual attention (Teoh et al., 2020). Here, we tested the idea that similar processes may explain the enhancement of framing biases under time constraint. Our argument rests on two observations that call important assumptions of dual-system models of the framing effect into question.

First, one of the key assumptions of dual-system models that have been applied to framing effects is that choice results from a competition between a rapid, obligatory, and frame-sensitive System 1 and a slower, volitional, and rational System 2 (De Martino et al., 2006; Diederich & Trueblood, 2018; Epstein, 1994; Kahneman & Frederick, 2007; Metcalfe & Mischel, 1999; Stanovich & West, 2000; but cf. Bago & De Neys, 2017; Evans, 2007, 2012; Pennycook et al., 2015). In this view, shortened deadlines for decision making simply interrupt this sequence of events, reducing System 2 engagement and increasing the influence of System 1. However, this view discounts a large body of research showing that decision makers adopt a variety of choice strategies to compensate for time restrictions (e.g., Miller, 1960; Payne et al., 1993). For example, decision makers may strategically and selectively deploy attention when time is limited—prioritizing information that is predictive of reward (Mitchell et al., 2012) or more efficient to process (e.g., pictures vs. text; Pieters & Warlop, 1999). Because attention may play an important role in risky choice (e.g., Fiedler & Glöckner, 2012; Pachur et al., 2018; Stewart et al., 2016) and time pressure may cause decision makers to adapt their information search (e.g., Payne et al., 1988; Teoh et al., 2020), we hypothesized that attentional shifts under time pressure could offer an alternative explanation for increased framing effects. In other words, time restrictions may change what information is processed and in what order this processing occurs because of strategic adaptations in visual search.

Our second observation is that dual-system models of framing effects have often assumed that System 1 processes stimuli in a parallel, holistic fashion, combining a large amount of information simultaneously (e.g., Diederich & Trueblood, 2018), and fail to address potentially relevant consequences of serial information search (see Gottlieb, 2012). To illustrate this point, consider Diederich and Trueblood’s (2018) dual-system computational model of the framing effect. In their model, System 1 rapidly computes a signal representing the difference in value between two options in which probabilities and framed outcomes have been weighted by parameters derived from prospect theory (Kahneman & Tversky, 1979). After some time, System 2 computes a value on the basis of normative expected value, which replaces System 1 inputs to evidence accumulation, resulting in more rational choice. Whereas this model is both elegant and computationally rigorous, it implies that several pieces of information (i.e., the probability and outcome information of each option) enter the evidence-accumulation process simultaneously. Yet if the limits of visual attention mean that each of these pieces of information must be attended sequentially before use, and if this information enters the evidence-accumulation process as it is attended, the serial order of visual attention could profoundly influence choices. First, *primacy effects* could occur if earlier information alters the extent to which later information influences behavior. Second, *gatekeeping effects* could occur if choices are made before all information has been attended (Orquin et al., 2018). Both effects may be particularly likely to happen under time constraints, when decision makers might prioritize making fast, rather than fully informed, choices (Teoh et al., 2020). Evidence already suggests that attending to framing information first increases framing biases (Kwak & Huettel, 2018). Whether this accounts for increases in framing under time pressure remains unknown.

**Statement of Relevance**

Psychological scientists have long studied the causes of people’s many seemingly irrational choice biases (e.g., the framing effect, in which superficial differences in the way a choice problem is described change people’s preferences). One influential theory has been that framing effects emerge from fast, automatic emotional responses that must be corrected by slower, deliberative thought. The observation that time pressure (which presumably disrupts controlled processing) amplifies framing effects seems to support this idea. Here, we provide evidence for an alternative explanation based on the reasoning that people must attend to choice attributes to use them, that attending to an attribute takes time, and that time pressure reduces the amount of information people can attend. In particular, by tracking eye gaze, we found that time pressure leads people to more strategically allocate their attention in a way that amplifies choice biases. Our findings suggest that rather than preventing rational thought from overriding emotion, time pressure may increase seemingly irrational behavior for rational and strategically adaptive reasons.

In our study (preregistered on OSF at https://osf.io/7j6kh/).^1^ we aimed to test whether time-pressure-induced amplification of the framing effect, which has previously been interpreted as evidence for dual systems, might instead be the result of adaptive-attentional shifts. To do so, we recorded eye-gaze position while decision makers completed a direct replication of an experiment in which time constraints amplified the framing effect (Guo et al., 2017). We tested two key predictions motivated by the above considerations: (a) that imposed time constraints would induce shifts in early attention allocation and (b) that changes to the order of attention allocation would amplify the framing effect. In addition to testing these two primary hypotheses, we explored how peripheral vision contributes to early attention biases and whether the mechanism by which ordered attention modulates the framing effect is more consistent with primacy, gatekeeping, or both.

## Method

### Participants

Participants were recruited from the University of Toronto Scarborough psychology-experiment participation pool and with flyers posted around campus. A university student population matches what was used in the experiments we were attempting to replicate (Guo et al., 2017). After preregistered exclusions (see below), the sample consisted of 40 participants (15 male; mean age = 19.48 years) for all analyses. For analyses that did not involve eye-tracking data, we included five additional participants who were excluded from eye-tracking analyses only because of poor eye-tracker calibration. Participants from the participation pool received course credit, whereas flyer-recruited participants received a base payment for their time. All participants earned a monetary bonus based on their task performance (see the Risky-Choice Task section), which ranged from $0 to $5 (Canadian).

#### Sample-size justification

We used the data from Experiment 1 by Guo et al. (2017) to determine a sample size for our experiment that would be sufficient to detect a true Frame × Time Constraint interaction on choice with a probability exceeding .80. According to our simulations of a 2 (frame) × 2 (time-constraint condition) repeated measures analysis of variance (ANOVA; Lakens & Caldwell, 2021), 40 participants would provide power of .98.

#### Participant-level exclusions

In total, 61 participants completed the experiment. As outlined in our preregistration, we applied a number of predetermined exclusion criteria. First, within participants, we excluded (a) any block in which the participant gave the same response on 90% or more of trials, (b) time-constraint blocks in which the participant failed to respond before the deadline on 25% or more of trials, (c) no-time-constraint blocks in which the participant had a response time of less than 500 ms on 10% or more of trials, and (d) any block in which eye-tracker calibration was poor (as determined by EyeLink software [SR Research, Mississauga, Ontario, Canada]; i.e., largest error > 2.0° or average error > 1.5°). We then excluded participants who (a) had fewer than two blocks of usable data for either time-constraint condition (three participants excluded because of behavioral criteria; six because of eye-tracking criteria), (b) chose the option with the lower expected value on more than 25% of catch trials (for a definition, see the Risky-Choice Task section) in the no-time-constraint blocks (14 participants; for a comparable exclusion rate based on this criterion, see Experiment 3 of Guo et al., 2017), (c) chose the same option (e.g., the gamble) on 90% or more of total trials (zero participants), (d) self-reported low English fluency (one participant), or (e) self-reported that their vision was currently uncorrected but that it usually needed to be (one participant).

### Procedure

All experimental procedures were approved by the Research Ethics Board at the University of Toronto and conducted in accordance with its guidelines. After arriving at the laboratory, participants provided informed consent and then received instructions regarding both the risky-choice task and eye-tracking procedures (e.g., stay as still as possible, always fixate on the cross when it appears) on a computer. As part of the instructions, participants were guided through four example trials of the risky-choice task. Following the instructions, participants completed a brief multiple-choice quiz over what they had read and were required to answer each question correctly before advancing. They then completed five practice no-time-constraint trials. After the practice no-time-constraint trials, the experimenter checked that participants understood the task and then verbally reviewed the eye-tracking procedures. Participants then completed the first no-time-constraint block. Next, participants completed five practice time-constraint trials before beginning the second block (i.e., the first time-constraint block). Participants were allowed to take breaks between each block of the task as desired. After completing all risky-choice task blocks, participants completed a speeded numerical-estimation task, a self-report survey about the risky-choice task, and several self-report personality measures (see preregistration for a complete list of measures). Participants were then debriefed, paid, and dismissed. Participants completed the experiment one at a time in individual sessions. All experimental materials were presented using PsychoPy (Version 3.2.3; Peirce et al., 2019).

### Risky-choice task

The main experimental task was modeled closely on the experiments conducted by Guo et al. (2017). For the target trials, a set of 72 endowment and probability combinations was generated. Starting-point endowments were drawn from a uniform distribution: *U*(20, 90). The probabilities of winning the gambles were drawn equally from each of three truncated normal distributions (*M*s = .28, .42, and .56; *SD*s = .2; limits = .1 and .9). For each endowment-probability pair, we created gain- and loss-framed sure options that matched the expected value of the gamble. For example, if the endowment was 60 points and the probability of winning was .25, then the sure option would be either “keep 15 points” (gain frame) or “lose 45 points” (loss frame). Participants were presented with each version (gain and loss) of a given endowment-probability pair once under both time-constraint conditions. Therefore, there were 288 target trials in total (72 endowment-probability pairs × 2 frames × 2 time-constraint conditions). The task also included 32 catch trials in which the expected values of the sure option and the gamble were significantly different (by 20–30 points); catch trials were excluded from all analyses. The starting-point endowments for the catch trials were drawn from the same distribution as the target trials. For half of the catch trials, the sure option had the higher expected value. The same catch trial was presented with each sure-option framing once per time-constraint condition.

**Fig. 1.**
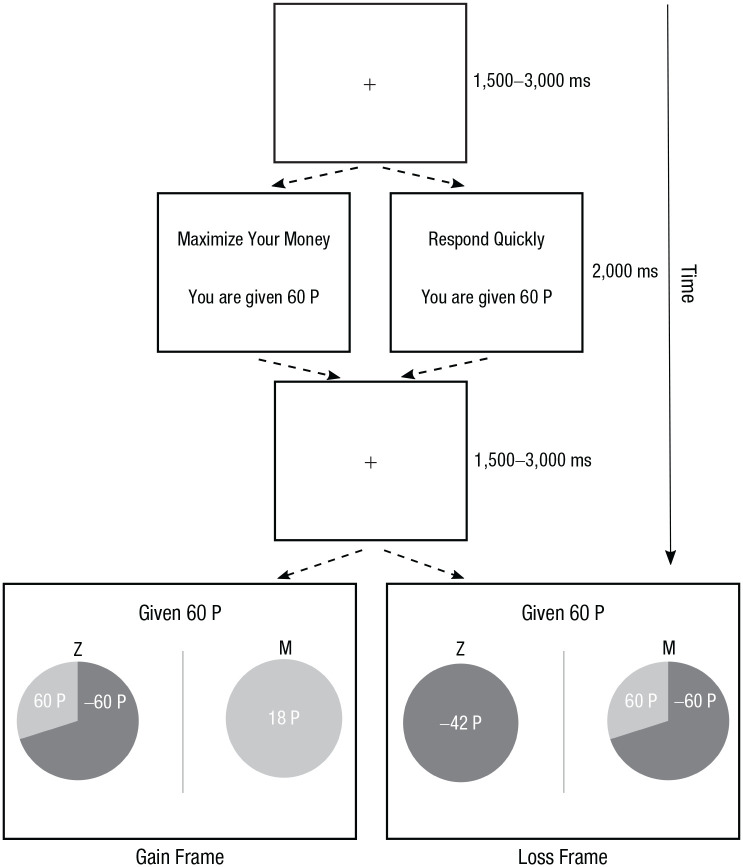
Design of the risky-choice task. At the beginning of each trial, participants made a brief fixation. Then they saw their initial endowment and a message corresponding to the time-constraint condition (“Maximize Your Money” during no-time-constraint blocks and “Respond Quickly” during time-constraint blocks). After another fixation cross, participants were presented with two options: one sure option and one gamble, shown in pie charts. The sure option depicted a certain outcome framed as either a gain (18 points [P] in the example trial on the left) or a loss (–42 P in the example trial on the right). The gamble gave some probability of either keeping or losing the entire endowment (e.g., a 70% chance of losing 60 P vs. a 30% chance of gaining or keeping 60 P). The two options were equated for expected value on all target trials, and participants indicated their choice by pressing the Z or M key. During no-time-constraint blocks, participants were given unlimited time to choose, whereas they had only 1 s to respond during time-constraint blocks. The side on which each option appeared was randomly varied. The shade of gray used to depict gains and losses was counterbalanced across participants. After the choice screen, participants received conditional feedback (see the Method section).

The 320 trials were divided into eight blocks of 40 trials each. During odd-numbered blocks, participants were told that they could make their choices at “[their] own pace” and should “try to maximize the money [they] earn.” During these blocks, there was no time limit by which decisions had to be submitted for each trial. Meanwhile, on even-numbered blocks, the amount of time that participants had to make each choice was restricted to 1,000 ms, and participants were told to “respond as quickly as [they] can while trying to maximize the money [they] earn.” On each trial of the risky-choice task, participants first saw a fixation cross for a uniformly random duration between 1,500 and 3,000 ms (see Fig. 1). Participants were instructed to always fixate on the cross when it appeared. Next, participants were presented with their starting-point endowment for that trial. This screen was displayed for 2,000 ms and also showed brief instructions about the current block (“Maximize Your Money” during no-time-constraint blocks and “Respond Quickly” during time-constraint blocks). Next, participants again saw a fixation cross for 1,500 to 3,000 ms. The choice options were then displayed. On each trial, the participant was given the choice between a sure option and a gamble, each presented as a pie chart depicting the probabilities of keeping or losing some amount of the initial endowment. Because the outcome depicted by the sure option always had a probability of 1.0, the pie chart for the sure option was always a single solid color, whereas the gamble pie chart was divided according to the probabilities of the outcomes. The probabilities of gaining/keeping or losing some proportion of the endowment were always represented with the same shades of gray (light or dark) for any individual participant, but which shade of gray was used to depict gains and which to depict losses was counterbalanced across participants. The side on which each option appeared varied randomly from trial to trial with the requirement that the sure option and gamble appeared on the left side for an equal number of trials in each of the four conditions (2 frames × 2 time constraints). To make their choice, participants pressed the Z key or M key to select the option on the left or right, respectively, at which point the choice options disappeared.

As in the study by Guo et al. (2017), we emphasized the goal of maximizing earnings during no-time-constraint blocks by presenting feedback (for 2,000 ms) after every trial stating how much had been earned on that trial. During time-constraint blocks, participants received no feedback if they responded in time. However, when a participant failed to respond before the deadline, feedback was presented for 2,000 ms stating that they did not earn any points because they did not respond in time. We chose to give participants choice feedback only in the no-time-constraint blocks in line with the procedure for the largest experiment conducted by Guo et al. (2017), but it is worth noting that several experiments have found the same effects of time constraint on the framing effect when giving identical feedback in both conditions (Diederich et al., 2020; Guo et al., 2017). Each block contained an equal number of gain- and loss-framed trials.

As in Experiment 2 by Guo et al. (2017), participants were instructed at the beginning of the task that they had to earn a minimum number of points in each block in order to keep them. Specifically, participants were instructed that they had to earn at least 1,050 points in a block to keep the points that had been earned during that block. Furthermore, they were informed that it would not be possible to earn a sufficient number of points by choosing the sure option on each trial. Participants were told how many points they had earned during each block at the end, whether this amount met the required amount, and how many total points they had so far accumulated.

### Eye tracking: acquisition, preprocessing, and analysis

Gaze position was recorded using an EyeLink 1000 Plus Desktop Mount eye tracker (SR Research, Mississauga, Ontario, Canada). The EyeLink system was configured using a 35-mm lens, monocular recording, and a sampling rate of 1,000 Hz. Participants rested their heads on a chin rest positioned 85 cm from the display monitor, which had a screen resolution of 1,920 × 1,080 pixels. The visual angle between the centers of the two pie charts for the choice options was approximately 13.79° (7.58° between the inner edges), and each pie chart subtended approximately 6.08° × 6.08°.

Before each of the eight blocks, a full 9-point gaze-location calibration and validation were conducted. Calibration and validation were repeated as needed to improve results. However, if the final validation before starting the block was poor, as determined by EyeLink software, that block was excluded from any analyses involving eye-tracking data, in line with our preregistered exclusion criteria. EyeLink’s online parser classified fixation, saccade, and blink events using gaze position with the *cognitive configuration* of EyeLink’s velocity- and acceleration-based algorithm. Specifically, saccades were identified with a motion threshold of 0.15°, velocity threshold of 30° per second, acceleration threshold of 8,000° per second squared, and a pursuit threshold of 60° per second. The raw fixation events (i.e., without filtering based on duration or merging) were exported from EyeLink’s DataViewer and preprocessed using custom R scripts. Drift was corrected off-line by first calculating the median fixation position during the two fixation crosses (pre- and postendowment screen) for each trial. If the distance between the median position and the center of the screen was greater than 100 pixels, then the fixations for that trial were shifted so that the median position during the fixation crosses was at the center of the screen. Next, 500- × 500-pixel areas of interest (AOIs) were defined around the choice options, which were 380 pixels in diameter, giving a 60-pixel margin on each side. Fixations were classified according to whether they fell inside either the sure-option AOI, gamble AOI, or neither. Next, consecutive fixations within the same AOI were merged if the interfixation time was 100 ms or less. For instance, if two consecutive fixations of 100 ms and 150 ms, both within the gamble AOI, were separated by 50 ms of missing fixation, they were merged into a single 300-ms fixation on the gamble. However, if they were separated by more than 100 ms, then they were not merged. Last, if none of the fixations composing a merger had been 100 ms or more, then that merged fixation was excluded. That is, there had to be at least one stable gaze for at least 100 ms to be considered a fixation.

In our preregistered exclusion criteria, we wrote that we intended to drop any trials from eye-tracking analyses in which the participant never fixated either choice option or in which gaze-position data were missing from 25% or more of the samples during the choice option screen. Because applying these criteria resulted in the exclusion of a sizable number of trials (an additional 19.95% of trials that remained after other exclusions), we modified our procedure to instead exclude from eye-tracking analyses any trials in which (a) the participant never fixated either choice option, (b) 35% or more of the samples had missing gaze-position data, and (c) there were 500 ms or more of consecutive time points with missing data. These modified exclusion criteria resulted in 8.89% of trials being excluded. All conclusions were the same with either set of exclusion criteria.

## Results

### Effects of time constraint on choice

**Fig. 2.**
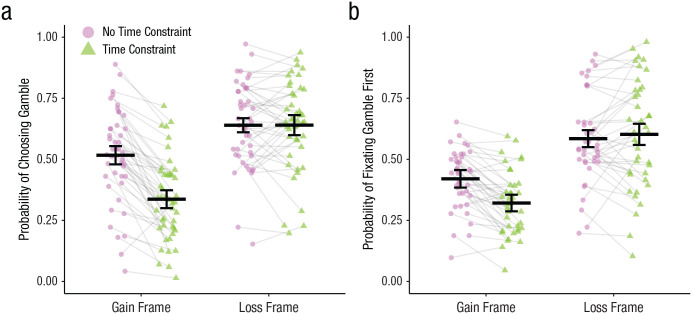
Probability of (a) choosing the gamble and (b) fixating the gamble first as a function of frame and time constraint. Lines between data points connect individual participant means in each time-constraint condition. Solid horizontal lines show means across each time-constraint condition, and error bars represent 95% confidence intervals with repeated measures corrections (Morey, 2008).

First, we verified that we replicated Guo et al.’s (2017) results by conducting a 2 (frame: gain, loss) × 2 (time constraint: none, 1 s) repeated measures ANOVA on the proportion of choices in which the gamble was selected. As expected, we found significant main effects of both frame, *F*(1, 44) = 76.00, *p* < .001, η_G_^2^ = .27, and time constraint, *F*(1, 44) = 34.74, *p* < .001, η_G_^2^ = .06, which were qualified by a significant Frame × Time Constraint interaction, *F*(1, 44) = 57.06, *p* < .001, η_G_^2^ = .06; the framing effect was larger when participants had limited time (gain: *M* = .34, *SD* = .17; loss: *M* = .64, *SD* = .18), *t*(44) = 10.18, *p* < .001, *d* = 1.52, 95% confidence interval (CI) = [1.08, 1.95], than when they had unlimited time (gain: *M* = .52, *SD* = .19; loss: *M* = .64, *SD* = .17), *t*(44) = 5.05, *p* < .001, *d* = 0.75, 95% CI = [0.42, 1.09], to make a decision (see Fig. 2a). Interestingly, in our study, the significant increase in the framing effect under time constraint was driven entirely by participants choosing the sure option more frequently when time was limited in gain-framed trials, *t*(44) = 8.83, *p* < .001, *d* = 1.32, 95% CI = [0.91, 1.72], whereas there was no effect of time on choices in loss-framed trials, *t*(44) = −0.02, *p* = .98, *d* = 0.003, 95% CI = [–0.29, 0.30]. Although this pattern of results has not been explicitly reported before, a reanalysis of results from past studies (i.e., Diederich et al., 2020; Guo et al., 2017) shows that the effect of time constraint on choice has been consistently weaker and sometimes absent in loss-framed trials (see Tables S1 and S2 in the Supplemental Material available online). Thus, our results replicate those of prior studies.

### Effects of Frame × Time Constraint interactions on early attention

First fixation. The dual-system interpretation of the above result is that time constraints caused decision makers to choose before rational expected values from System 2 could override biased, framing-sensitive values from System 1. However, our attentional account predicts that decision makers likely modify important aspects of their choice, such as early attentional allocation, which could result in a systematic change to the information that is initially processed. To test this possibility, we conducted a 2 (frame) × 2 (time constraint) repeated measures ANOVA on the proportion of trials in which the gamble was fixated first (see Fig. 2b). Consistent with predictions, results mirrored the choice patterns above, revealing main effects of both frame, *F*(1, 39) = 74.32, *p* < .001, η_G_^2^ = .31, and time constraint, *F*(1, 39) = 9.09, *p* = .005, η_G_^2^ = .01, which were qualified by a significant interaction, *F*(1, 39) = 20.63, *p* < .001, η_G_^2^ = .03. Unpacking the interaction revealed that when participants were given unlimited time, they were more likely to fixate the sure option first on gain-framed trials (*M* = .42, *SD* = .12) than loss-framed trials (*M* = .58, *SD* = .18), *t*(39) = −6.07, *p* < .001, *d* = 0.96, 95% CI = [0.58, 1.34]. This difference between gain-framed trials (*M* = .32, *SD* = .13) and loss-framed trials (*M* = .60, *SD* = .22) was amplified when decision time was restricted, *t*(39) = −9.21, *p* < .001, *d* = 1.46, 95% CI = [1.0, 1.9]. As with choices, the interaction between sure-option framing and time constraint was driven by an increased tendency to fixate the sure option first in gain-framed trials, *t*(39) = 6.54, *p* < .001, *d* = 1.03, 95% CI = [0.64, 1.42], whereas there was no effect in loss-framed trials, *t*(39) = −0.82, *p* = .42, *d* = 0.13, 95% CI = [–0.19, 0.45]. These results show clear evidence that decision makers’ early attention is systematically biased by the sure option’s framing and that this bias is magnified when time is limited in a way that mirrors their choices.

#### Effects of peripheral vision on early attention

The fact that first fixations were systematically influenced by the framing of the sure option, whose display position was unpredictable across trials, demonstrates that decision makers likely received some initial identifying information via their peripheral vision. In a preregistered prediction, we anticipated that decision makers would peripherally distinguish the options on the basis of the colors of the pie charts. Visual attention is attracted to stimuli that are predictive of reward (Le Pelley et al., 2016), and the pie charts for sure gains and losses were colored with corresponding shades of gray. Sensitivity to these cues could account for the greater early attention to the sure option and gamble on gain- and loss-framed trials, respectively. Additionally, because the proportion of the gamble that was “gain colored” matched the probability of winning, we also predicted that first fixation would be sensitive to the gamble probability. For instance, it should be easier to distinguish a sure gain from a low-probability gamble than a high-probability gamble. Therefore, we predicted that the gamble’s probability of winning would be positively associated with the probability of fixating the gamble first and that this relationship would be stronger when time was limited.

**Fig. 3.**
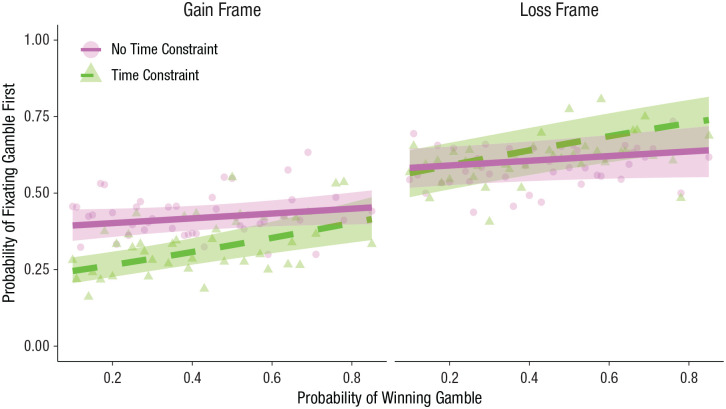
Probability of fixating the gamble first as a function of probability of winning the gamble and time constraint, separately for the gain and loss frames. Lines indicate best-fitting mixed-effects regressions (see the Method section), and shaded regions represent 95% confidence intervals. Dots and triangles depict means for each unique gamble probability.

To test this prediction, we first fitted a mixed-effects logistic regression predicting the probability of fixating the gamble first using frame, time constraint, probability of winning the gamble, and their interactions. The model also included a random intercept for participant and random slopes for frame, time constraint, and probability. Because the three-way interaction was nonsignificant, *b* = 0.51, *SE* = 0.49, *z* = 1.05, *p* = .29, we removed this term and other higher order interactions that did not significantly improve model fit. The final model had two interaction terms (Frame × Time Constraint and Probability × Time Constraint) and their simple effects (see Table S3 in the Supplemental Material). As reported above, there was a significant Frame × Time Constraint interaction, *b* = −0.62, *SE* = 0.09, *z* = −7.02, *p* < .001. Critically, there was also a significant Probability × Time Constraint interaction on first fixation, *b* = 0.72, *SE* = 0.24, *z* = 2.93, *p* = .003; participants were significantly more likely to fixate high-probability gambles first when time was limited, *b* = 1.04, *SE* = 0.21, *z* = 4.86, *p* < .001, but not when it was unlimited, *b* = 0.32, *SE* = 0.20, *z* = 1.62, *p* = .11 (see Fig. 3). Furthermore, as the probability of winning the gamble approached 1.0 for gain-framed trials and .0 for loss-framed trials, the color of the gamble more closely matched the corresponding sure option, and first fixation approached chance levels. These results suggest that participants used peripheral cues to direct initial attention, particularly under time constraints.

### Effects of Attention × Time Constraint interactions on choice

Having provided support for our first key hypothesis (that decision makers deploy attention strategically under time pressure), we next examined our second prediction that the attentional shift under time constraint explains the increased framing effect. The specific nature of the effect could take two forms. On the basis of past research (Kwak & Huettel, 2018), we preregistered the hypothesis that allocating early attention to the sure option under time constraints would be associated with an increase in framing-effect-consistent choices (i.e., risk averse for gain-framed trials and risk seeking for loss-framed trials). The rationale for this prediction draws on the fact that the sure option changes form under different frames, whereas the gamble is presented identically across conditions (see Fig. 1). Therefore, we expected a three-way Frame × Time Constraint × Attention interaction on the probability of choosing the gamble. However, an alternative (post hoc) hypothesis is that decision makers will be biased to choose whatever they are currently attending regardless of frame, particularly under time constraints (e.g., Stewart et al., 2016). This competing prediction anticipates a two-way Time Constraint × Attention interaction but no moderation by framing.

We fitted a mixed-effects logistic regression predicting the choice to gamble with dummy-coded frame, time constraint, first fixated option, and their interactions (see Table S4 in the Supplemental Material). A random intercept for participant and random slopes for frame, time constraint, and first fixation were included in the model. Contrary to our preregistered hypothesis, results for the three-way Frame × Time Constraint × First Fixation interaction were nonsignificant, *b* = 0.20, *SE* = 0.19, *z* = 1.05, *p* = .294. However, unpacking the model revealed results consistent with our post hoc alternative prediction: The Time Constraint × First Fixation interaction was significant and the same for both frames—gain: *b* = 0.64, *SE* = 0.14, *z* = 4.62, *p* < .001; loss: *b* = 0.44, *SE* = 0.14, *z* = 3.16, *p* = .002. When time was limited, fixating the gamble first increased the probability of choosing the gamble in both gain-framed trials, *b* = 0.82, *SE* = 0.12, *z* = 6.95, *p* < .001, and loss-framed trials, *b* = 0.47, *SE* = 0.12, *z* = 4.09, *p* < .001. Meanwhile, when time was unlimited, fixating the gamble first had no effect on choice—gain: *b* = 0.18, *SE* = 0.11, *z* = 1.71, *p* = .087; loss: *b* = 0.04, *SE* = 0.11, *z* = 0.34, *p* = .734. Thus, early attention had a larger impact on choice when time was limited than when it was unlimited; specifically, decision makers were more likely to choose the option they fixated first. Because initial attention was influenced by the sure-option framing, especially under time constraints, this resulted in a larger framing effect.

#### Gatekeeping versus primacy effects of attention

**Fig. 4.**
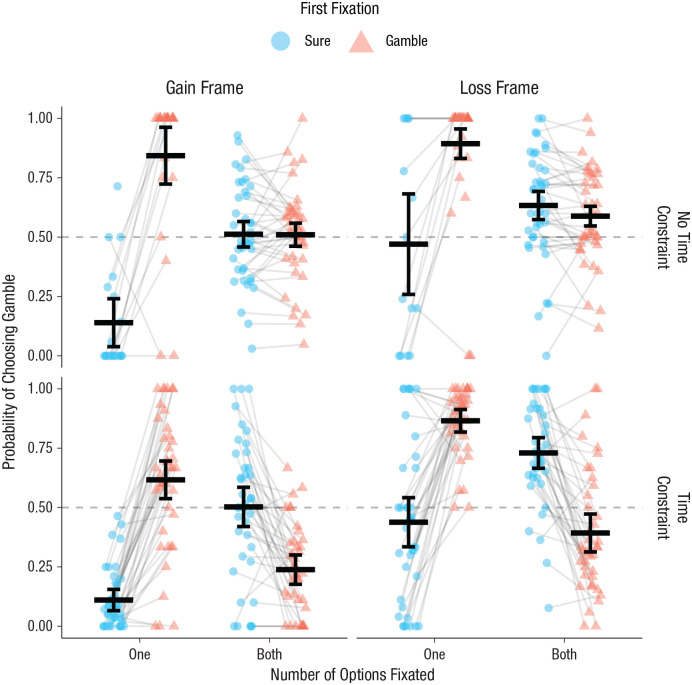
Probability of choosing the gamble as a function of number of options fixated and whether the sure option or the gamble was fixated first. Results are shown separately for each combination of time constraint and frame. Lines between data points connect individual participant means in each first-fixation condition. Solid horizontal lines show means across each first-fixation condition, and error bars represent 95% confidence intervals with repeated measures corrections (Morey, 2008). The dashed line indicates chance.

The above analysis leaves it unclear whether the effects of participants’ early attention on behavior were due to gatekeeping, primacy, or both. Testing these two mechanisms requires examining whether first fixations influence behavior only if choices are made before all options are attended (gatekeeping) or also influence choice even when all options are known. We thus introduced a dummy-coded variable indicating whether the participant had fixated both options or only one option on a given trial. An initial fit revealed that there was no four-way interaction of Frame × Time Constraint × First Fixation × Number of Options Fixated, so it was removed from the model. After simplifying the model by iteratively removing the remaining nonsignificant three-way interactions that did not improve fit, we arrived at a model with frame, time constraint, first fixation, number of options fixated, and all their possible two-way interactions as well as the three-way interaction of time constraint, first fixation, and number of options fixated (see Table S5 in the Supplemental Material). We also included a random intercept for participant and random slopes for frame, time constraint, first fixation, and number of options fixated. This exploratory model revealed a marginal three-way Time Constraint × First Fixation × Number of Options Fixated interaction, *b* = −0.68, *SE* = 0.36, *z* = −1.91, *p* = .056 (see Fig. 4).

When participants fixated only one of the two options, they were highly likely to choose whichever option they fixated regardless of whether time was unlimited, *b* = 3.52, *SE* = 0.32, *z* = 10.86, *p* < .001, or constrained, *b* = 2.95, *SE* = 0.15, *z* = 20.18, *p* < .001 (there was no difference between conditions, *b* = −0.57, *SE* = 0.34, *z* = −1.70, *p* = .09). Thus, there was evidence of a strong gatekeeping effect, independent of time constraints; specifically, decision makers rarely chose an option they had not seen.

Meanwhile, when participants fixated both of the options, there was a significant Time Constraint × First Fixation interaction, *b* = −1.25, *SE* = 0.13, *z* = −9.74, *p* < .001. First fixation had no effect on choice when time was unlimited and both options were attended, *b* = −0.17, *SE* = 0.09, *z* = −1.76, *p* = .08. However, fixating the gamble first and then the sure option actually reduced the chance of choosing the gamble when time was limited, *b* = −1.42, *SE* = 0.13, *z* = −11.29, *p* < .001. Thus, we found no evidence of a primacy effect. Instead, when participants looked at both options under time constraints, they tended to choose the opposite of whatever option they fixated first, a result that we will explore below.

The above results provide strong evidence for gatekeeping and little evidence of a primacy effect. Additional analysis showed that participants made a choice after just a single fixation on 54.4% of time-constraint trials (number of fixations: *M* = 1.53, *SD* = 0.21) compared with 11.7% of no-time-constraint trials (*M* = 4.77, *SD* = 2.07). Thus, gatekeeping played a role on more than 4 times as many trials when time was limited. These results demonstrate that, as emphasized by dual-system models, a strategy used by decision makers to meet the time limit was to truncate information search. Nevertheless, consistent with our visual attention explanation, results showed that for the gatekeeping mechanism to produce an amplified framing effect, the first fixation would have to differ depending on the framing of the sure option. The combined result of these two attentional effects is an amplified framing effect.

#### Effects of time constraint and expected value on information search

The observation that decision makers tended to choose the opposite of their first fixation (i.e., the second fixated option) when both options had been fixated under time restrictions was unpredicted. Because time-pressured participants frequently made their choice after fixating only one option, we made the post hoc prediction that participants adopted a satisficing strategy in which they first examined one option and immediately accepted it if it met some minimal value. However, if the first option was not satisfactory, they were more likely to fixate the other option. This indicates that the expected value of the first-fixated option should predict whether participants fixate one or both options under time constraint.

**Fig. 5.**
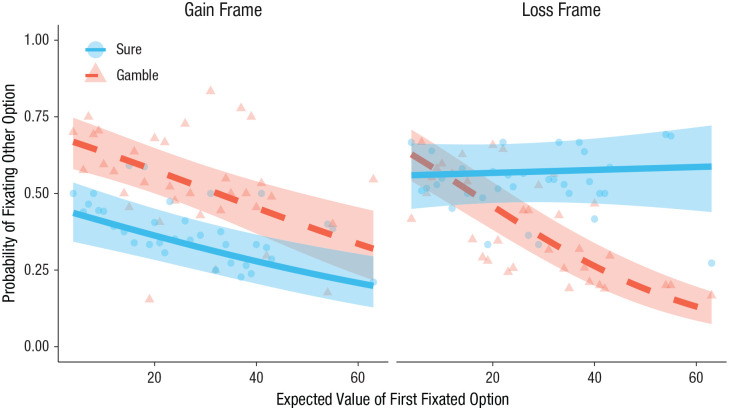
Probability of shifting attention to the second, unattended option as a function of expected value of the first fixated option and which option was fixated first in time-constraint trials, separately for the gain and loss frames. Lines indicate best-fitting mixed-effects regressions (see the Method section), and shaded regions represent 95% confidence intervals. Dots and triangles depict means for each unique expected value.

To test this possibility, we restricted our analysis to just the time-constraint trials and fitted a mixed-effects logistic regression with the expected value of the first fixated option (rescaled by dividing by 100 and then mean-centered), frame, first fixated option, and their interactions predicting whether participants proceeded to fixate the second option (see Table S6 in the Supplemental Material). Random slopes were included for participant as well as for frame and first fixated option (a random slope for expected value did not improve fit). This model revealed a significant three-way interaction, *b* = 3.97, *SE* = 1.05, *z* = 3.78, *p* < .001 (see Fig. 5). Examining gain-framed trials, we found that participants were significantly less likely to look at the second option if the first fixated option had a high expected value, regardless of whether they fixated the gamble, *b* = −2.47, *SE* = 0.58, *z* = −4.26, *p* < .001, or the sure option, *b* = −1.93, *SE* = 0.46, *z* = −4.18, *p* < .001. There was no First Fixation × Expected Value interaction, *b* = −0.54, *SE* = 0.74, *z* = −0.72, *p* = .47. Meanwhile, on loss-framed trials, there was a significant interaction, *b* = −4.51, *SE* = 0.75, *z* = −6.03, *p* < .001; participants were less likely to look at the second option if they first fixated a gamble with high expected value, *b* = −4.31, *SE* = 0.49, *z* = −8.74, *p* < .001, whereas there was no effect of expected value if they first fixated the sure loss, *b* = 0.20, *SE* = 0.56, *z* = 0.35, *p* = .73. In sum, there was clear evidence that the continuation of information search was influenced by the value of the option that was seen first in all cases except for when a sure loss was fixated first.

## Discussion

Challenging the dominant dual-system explanation of amplified framing effects under time constraint, we found strong evidence that time pressure induced pronounced attentional shifts that magnify the framing effect. When faced with a rapidly impending deadline, decision makers modified information search in at least two ways: (a) increased use of peripheral visual cues to guide early attention and (b) more frequent termination of information search after attending to only one option. On time-pressured, gain-framed trials, the combined effect of fixating sure gains first and deciding before looking at the gamble produced a significant choice shift. Finally, we found evidence that, under time constraints, the value of the first fixated option influenced the probability of continuing information search, suggesting a satisficing choice rule. Overall, our results present an important challenge to the dual-system account of the framing effect and have important implications for researchers using time constraints to make inferences about the operation of fast and slow systems.

Our attentional mechanism may explain a hitherto unremarked asymmetry in the influence of time pressure on framing biases. Our results, as well as a closer reading of past studies (Diederich et al., 2020; Guo et al., 2017; see Tables S1 and S2), suggest a reliably larger effect of time pressure on choice under gain than loss frames. Although existing dual-system models (e.g., Diederich & Trueblood, 2018) may be capable of explaining such an asymmetry by proposing differing delays before System 2 engagement between gain and loss framings, this raises additional questions about the source of this differential delay. Meanwhile, an adaptive-attention account may offer alternative, straightforward explanations. Indeed, the fact that limiting time did not influence either early attention or choice on loss-framed trials bolsters a causal interpretation of attention on choice and potentially the observed asymmetry. Thus, from an adaptive-attention perspective, the question becomes why time pressure induced early attention shifts for gain trials but not loss trials. Our data suggest that one possible contributing factor could be the distribution of gamble probabilities, which were skewed such that lower probabilities were more frequent (as in Experiments 1 and 2 by Guo et al., 2017). Because sure losses and low-probability gambles are more difficult to discriminate on the basis of peripheral cues, this feature of our task could have diminished the impact of time pressure on loss trials. The distinguishability of peripheral cues may, in turn, interact with other subtle task features such as the spatial distance between options. There may be other, non-vision-related factors that also contribute to the asymmetrical impact of time pressure, but our adaptive-attention account suggests that researchers in future work should carefully consider how subtle features of a choice task might interact with mechanisms of visual attention to influence behavioral effects.

Our results may also help to explain inconsistencies in the past literature. Although our study and others (Diederich et al., 2018, 2020; Guo et al., 2017) found that time limits magnified the framing effect, other studies have found the opposite (Igou & Bless, 2007; Kocher et al., 2013; Svenson & Benson, 1993). Furthermore, theoretical modulators of System 2 engagement such as critical thinking (LeBoeuf & Shafir, 2003; Smith & Levin, 1996), task motivation (Igou & Bless, 2007; Krishnamurthy et al., 2001), and cognitive load (Whitney et al., 2008) have produced similarly inconsistent results. Although these conflicting findings are difficult to reconcile with a dual-system model in which quick affective responses are the primary driver of framing effects, an adaptive decision-making framework that accounts for mechanisms such as visual attention may prove more useful. Different attentional models of risky choice predict both increased (Pachur et al., 2018) and decreased (Lieder et al., 2018) framing effects under time constraints. Exploring the different predictions of these models within specific decision contexts could bring greater clarity. For example, time constraints differentially influence attention to text and pictures (Pieters & Warlop, 1999). This could explain why time pressure has increased framing biases in studies with pictorial information (Diederich et al., 2018, 2020; Guo et al., 2017) but reduced them when more text-heavy methods are used (Igou & Bless, 2007; Svenson & Benson, 1993). In this way, choice biases such as the framing effect may be understood as emergent properties of an interaction between goal-directed decision strategies and features of the environment (Todd & Gigerenzer, 2020).

Just as attention’s role in producing framing effects is likely to vary across experimental paradigms, time pressure’s influence on attention and framing effects may differ between populations. Indeed, there is some past work showing differences in framing susceptibility as a function of age (Kim et al., 2005; Mikels & Reed, 2009) and sex (Fagley et al., 2010)—though sex did not moderate framing effects or attention in our experiment. However, far fewer studies have explored whether time pressure’s effects on decision making vary with age, sex, or culture. By highlighting the malleability of the choice process, our results point to the need for more research exploring how different people, including those from less Western, educated, industrialized, rich, and democratic samples (Henrich et al., 2010), may come to adopt different strategies in the face of time constraints (Miller, 1960; Payne et al., 1993).

Our findings may shed new light on neuroimaging results that have been interpreted as evidence in favor of dual systems. For example, the magnitude of the framing effect correlates with amygdala activation, which researchers have taken as evidence that framing effects rely on affect-based, System 1 processes (De Martino et al., 2006). However, other research implicates the amygdala in directing attention toward motivationally relevant stimuli (Cunningham & Brosch, 2012). Given this, we speculate that trial-level amygdala activation reflects enhanced attentional deployment to motivationally prioritized information (e.g., reward-predicting cues) and thus framing-effect-consistent choice. Amygdala involvement may also point to a mechanism by which time constraints modify attention. Time constraints increase decision makers’ anxiety (Kocher et al., 2013), which has been linked to increases in amygdala sensitivity (van Marle et al., 2009), attention to motivationally relevant information (Motoki et al., 2019), and framing susceptibility (Xu et al., 2013). Researchers should combine neuroimaging with eye tracking in future work to investigate the combined effects of attention and neural processes on the framing effect.

Our results also reinforce recent critiques that highlight the complexity of using decision speed to infer dual systems (Hutcherson et al., 2015; Krajbich et al., 2015). If manipulations of decision time induce strategic choice adaptations, they may be less capable of informing some dual-system models than previously believed (but see Bago & De Neys, 2017; Pennycook et al., 2016). That is, time constraints may violate assumptions of internal validity because they do not produce one single change in the choice process (Roberts & Hutcherson, 2019). Anticipating resource limitations such as time pressure, a decision maker may make multiple compensatory changes to their choice strategy using known features of the environment to ameliorate performance. What patterns result from this process may vary depending on context. Thus, whereas attentional shifts played a significant role in our paradigm, which features informative peripheral visual cues, attention may play a diminished or different role in other choice contexts depending on the advantages that it confers (e.g., single-shot, text-based paradigms). These insights suggest that researchers should avoid treating all framing effects (and their underlying drivers) as the same.

Finally, we note that our results do not necessarily dictate that dual-system models more broadly must be abandoned or that there is no role for factors such as automaticity in producing framing biases. Attentional processes and dual systems may coexist, necessitating the development of a hybrid model that specifies what aspects of choice result from discrete automatic and controlled systems and how such processes interact with adaptive information-processing strategies. For instance, System 2 processes involving working memory could be responsible for selecting the strategy of shifting attention under time pressure (Evans & Stanovich, 2013; Kiyonaga & Egner, 2013; Markovits et al., 2013). Such a model would flip the traditional dual-system account of framing effects on its head by suggesting that their amplification under time constraint is actually the result of an *increased* influence of System 2. Furthermore, whereas our findings clearly indicate a crucial role for visual attention, there are likely additional mechanisms that contribute to the formation of the framing effect. Thus, whether a full depiction of the framing effect requires an underlying dual-system architecture or can be explained with a single attention-guided, information-sampling process (e.g., Song et al., 2019) will need to be investigated in future work, especially in conjunction with computational models of the behavior-generating process. It will be up to the ingenuity of researchers to unpack the complex processes that underlie the extreme adaptability of human behavior.

## Supplemental Material

sj-pdf-1-pss-10.1177_09567976211026983 – Supplemental material for Time to Pay Attention? Information Search Explains Amplified Framing Effects Under Time Pressure Supplemental material, sj-pdf-1-pss-10.1177_09567976211026983 for Time to Pay Attention? Information Search Explains Amplified Framing Effects Under Time Pressure by Ian D. Roberts, Yi Yang Teoh and Cendri A. Hutcherson in Psychological Science

## Transparency

*Action Editor:* Leah Somerville

*Editor:* Patricia J. Bauer

*Author Contributions*

I. D. Roberts and C. A. Hutcherson developed the study concept. All the authors contributed to the study design and preregistration. I. D. Roberts collected and analyzed the data and wrote the initial draft of the manuscript. C. A. Hutcherson and Y. Y. Teoh provided critical revisions. All the authors approved the final manuscript for submission.

**Open Practices:** All data, materials, and analysis code have been made publicly available via OSF and can be accessed at https://osf.io/7j6kh/. The design and analysis plans were preregistered on OSF at https://osf.io/7j6kh/. Note that although the preregistration was uploaded prior to data collection, it was not registered at that time; however, as the time stamp shows, no changes were made to the preregistration after it was initially posted. Deviations from the preregistration are discussed in the text, and the Supplemental Material available online includes a section describing how the preregistered hypotheses map onto the analyses reported in the article. This article has received the badges for Open Data, Open Materials, and Preregistration. More information about the Open Practices badges can be found at http://www.psychologicalscience.org/publications/badges.

## Notes

1. Our preregistration outlined our hypotheses in a hierarchical fashion. In this article, we report our findings for analyses testing our primary hypotheses, a few tentative hypotheses, and a few post hoc hypotheses. For the sake of space, concision, and clarity, we do not include results for all our preregistered tentative and exploratory hypotheses in this article.
