# Supplementary material for: Time to Pay Attention? Information Search Explains Amplified Framing Effects Under Time Pressure
Source: Psychol Sci. 2021 Dec 3;33(1):90–104. doi: 10.1177/09567976211026983 (PMC8985223; doi:10.1177/09567976211026983)
Supplement: sj-pdf-1-pss-10.1177_09567976211026983 – Supplemental material for Time to Pay Attention? Information Search Explains Amplified Framing Effects Under Time Pressure [file sj-pdf-1-pss-10.1177_09567976211026983.pdf]

SUPPLEMENTAL MATERIAL

Time to pay attention?: Information search explains amplified framing effects under time pressure

Ian D. Roberts[a,1], Yi Yang Teoh[a], and Cendri A. Hutcherson[a,b]

[a]Department of Psychology, University of Toronto, 1265 Military Trail, Toronto, Ontario M1C 1A4, Canada

[b]Rotman School of Management, University of Toronto

[1]To whom correspondence should be addressed. Email to iandavidroberts@gmail.com

Table of Contents

REANALYSIS OF GUO ET AL. (2017) & DIEDERICH ET AL. (2020) 3

TABLE S1. FRAMING × TIME PRESSURE ON FRAMING-EFFECT-CONSISTENT CHOICE 4

TABLE S2. FRAMING × TIME PRESSURE ON FRAMING-EFFECT-CONSISTENT CHOICE SIMPLE EFFECTS 5

FULL MODEL DETAILS 6

TABLE S3. CONDITION, FRAME, AND GAMBLE PROBABILITY PREDICTING FIRST FIXATION 6

TABLE S4. CONDITION, FRAME, AND FIRST FIXATION PREDICTING CHOICE 7

TABLE S5. CONDITION, FRAME, FIRST FIXATION, AND NUMBER OF OPTIONS FIXATED PREDICTING CHOICE 8

TABLE S6. FRAME, FIRST FIXATION, AND EXPECTED VALUE OF FIRST FIXATED OPTION PREDICTING FIXATING THE SECOND OPTION UNDER TIME CONSTRAINTS 9

LINKAGES BETWEEN ANALYSES AND PREREGISTRATION 10

TABLE S7. MAPPINGS BETWEEN REPORTED ANALYSES AND PREREGISTERED HYPOTHESIS 11

REFERENCES 12

Reanalysis of Guo et al. (2017) & Diederich et al. (2020)

As reported in the main article, the significant increase in the framing effect under time constraint in our study was driven entirely by a change in choice for gain-framed trials – there was no effect of time pressure on choices for the loss-framed trials. Although this pattern of results has not been explicitly reported before, a re-analysis of past results from the studies on which ours was based (i.e., Diederich et al., 2020; Guo et al., 2017) shows that the effect of time constraints on risky monetary choices has been consistently weaker and sometimes absent for loss framed trials.

For our re-analysis, we compared the effect of time pressure on the tendency to make framing-effect-consistent choices for gain- versus loss-framed trials, by flipping how choices were coded for gain-framed trials. Thus, a higher value for the dependent variable meant increased probability of choosing the sure option for gain-framed trials and the gamble for loss-framed trials – that is, increased probability of choosing the framing-effect-consistent option. For experiment 1 of Guo et al. (2017), we collapsed across the different variations. For data from Diederich et al. (2020), we collapsed across the need and order conditions. A 2 (time pressure) × 2 (framing) repeated-measures ANOVA of the combined data from these 5 experiments revealed a significant time pressure × frame interaction such that the effect of time pressure was larger for gain-framed than for loss-framed trials (see Table S1). Furthermore, unpacking this interaction (see Table S2) suggests that the magnitude of the effect of time pressure on framing-effect-consistent choice is approximately twice as large for gain-framed (Cohen's $d = 0.51$) as opposed to loss-framed trials ($d = 0.25$), according to past studies.

Table S1. Framing × Time Pressure on Framing-Effect-Consistent Choice

	Experiment	N	Effect	F	p	η^2_G
Guo et al. (2017)	1	195	Frame	0.69	.410	.002
			Time Pressure	78.02	< .001	.025
			Frame × Time Pressure	2.50	.115	.002
	2	13	Frame	2.60	.133	.167
			Time Pressure	5.47	.038	.004
			Frame × Time Pressure	2.12	.171	.001
	3	52	Frame	37.70	< .001	.316
			Time Pressure	22.92	< .001	.033
			Frame × Time Pressure	1.96	.167	.003
Diederich et al. (2020)	1	19	Frame	18.38	< .001	.447
			Time Pressure	73.52	< .001	.057
			Frame × Time Pressure	12.63	.002	.023
	2	54	Frame	46.58	< .001	.370
			Time Pressure	13.42	< .001	.013
			Frame × Time Pressure	0.39	.536	.0004
Guo et al. & Diederich et al. Combined		333	Frame	13.13	< .001	.026
			Time Pressure	123.51	< .001	.021
			Frame × Time Pressure	5.52	.019	.002
Roberts et al.		45	Frame	2.41	.128	.035
			Time Pressure	57.06	< .001	.062
			Frame × Time Pressure	34.74	< .001	.061

Table S2. Framing × Time Pressure on Framing-Effect-Consistent Choice Simple Effects

Mean and standard deviations for framing-effect-consistent choice within each time pressure × frame cell. Paired t-test compares choice under high versus no/low time pressure for each framing condition.

			Time Pressure				
	Experiment	Frame	None / Low	High	*t*	*p*	*d*
Guo et al. (2017)	1	Gain	0.59 (0.24)	0.69 (0.23)	7.17	< .001	0.51
		Loss	0.59 (0.24)	0.65 (0.27)	3.65	< .001	0.26
	2	Gain	0.46 (0.21)	0.51 (0.20)	3.79	.003	1.05
		Loss	0.66 (0.22)	0.67 (0.23)	0.61	.553	0.17
	3	Gain	0.42 (0.11)	0.49 (0.15)	4.08	< .001	0.57
		Loss	0.62 (0.13)	0.65 (0.14)	2.34	.023	0.32
Diederich et al. (2020)	1	Gain	0.42 (0.14)	0.53 (0.13)	7.66	< .001	1.76
		Loss	0.71 (0.15)	0.74 (0.14)	1.80	.089	0.41
	2	Gain	0.46 (0.14)	0.50 (0.15)	2.74	.008	0.37
		Loss	0.69 (0.15)	0.72 (0.15)	2.42	.019	0.33
Guo et al. & Diederich et al. Combined		Gain	0.53 (0.22)	0.61 (0.22)	9.27	< .001	0.51
		Loss	0.62 (0.21)	0.67 (0.23)	4.63	< .001	0.25
Roberts et al.		Gain	0.48 (0.19)	0.66 (0.17)	8.83	< .001	1.32
		Loss	0.64 (0.17)	0.64 (0.18)	0.02	.982	0.003

Full Model Details

Table S3. Condition, Frame, and Gamble Probability Predicting First Fixation

Mixed-effects logistic regression predicting fixating the gamble first. Time constraint condition was dummy-coded (NTC = 0, TC = 1) and framing was effects-coded (gain = 1, loss = -1). Participants were treated as a random effect with varying intercepts ($s^2 = 0.32$, SD = 0.56) and varying slopes for condition ($s^2 = 0.10$, SD = 0.14), frame ($s^2 = 0.14$, SD = 0.37), and gamble probability ($s^2 = 0.49$, SD = .70).

Parameter	Estimate	SE	*z*	*p*
Intercept	-0.08	0.11	-0.70	.48
Condition	-0.45	0.12	-3.87	< .001
Gamble Probability	0.32	0.20	1.62	.11
Frame	-0.38	0.07	-5.80	< .001
Condition × Gamble Probability	0.72	0.24	2.93	.003
Condition × Frame	-0.31	0.04	-7.02	< .001

Table S4. Condition, Frame, and First Fixation Predicting Choice

Mixed-effects logistic regression predicting choosing the gamble. Time constraint condition (NTC = 0, TC = 1), framing (gain = 0, loss = 1), and first fixation (sure = 0, gamble = 1) were dummy-coded. Participants were treated as a random effect with varying intercepts ($s^2 = 0.91$, SD = 0.95) and varying slopes for condition ($s^2 = 0.14$, SD = 0.38), frame ($s^2 = 0.60$, SD = 0.76), and first fixation ($s^2 = 0.11$, SD = .33).

Parameter	Estimate	SE	*z*	*p*
Intercept	-0.07	0.16	-0.42	.68
Condition	-1.09	0.11	-10.15	< .001
Frame	0.69	0.16	4.40	< .001
First Fixation	0.18	0.11	1.71	.087
Condition × Frame	0.69	0.14	5.03	< .001
Condition × First Fixation	0.64	0.14	4.62	< .001
Frame × First Fixation	-0.14	0.13	-1.08	.28
Condition × Frame × First Fixation	-0.20	0.19	-1.05	.29

Table S5. Condition, Frame, First Fixation, and Number of Options Fixated Predicting Choice

Mixed-effects logistic regression predicting choosing the gamble. Time constraint condition (NTC = 0, TC = 1), framing (gain = 0, loss = 1), first fixation (sure = 0, gamble = 1), and number of options fixated (one = 0, both = 1) were dummy-coded. Participants were treated as a random effect with varying intercepts (s^2 = 1.09, SD = 1.04) and varying slopes for condition (s^2 = 0.17, SD = 0.41), frame (s^2 = 0.49, SD = 0.70), first fixation (s^2 = 0.15, SD = .39), and number of options fixated (s^2 = 0.27, SD = .52).

Parameter	Estimate	SE	*z*	*p*
Intercept	-1.60	0.28	-5.75	< .001
Condition	-0.72	0.24	-2.97	.003
First Fixation	3.60	0.33	11.04	< .001
Options Fixated	1.67	0.24	6.85	< .001
Frame	1.39	0.20	7.08	< .001
Condition × First Fixation	-0.57	0.33	-1.70	.089
Condition × Options Fixated	0.71	0.25	2.90	.004
First Fixation × Options Fixated	-3.68	0.33	-11.33	< .001
Condition × Frame	0.27	0.12	2.28	.022
First Fixation × Frame	-0.17	0.11	-1.58	.11
Options Fixated × Frame	-0.82	0.15	-5.58	< .001
Condition × First Fixation × Options Fixated	-0.68	0.36	-1.91	.056

Table S6. Frame, First Fixation, and Expected Value of First Fixated Option Predicting Fixating the Second Option under Time Constraints

Mixed-effects logistic regression predicting choosing the gamble. Expected value of the first fixated option was rescaled by dividing by 100 and then mean-centered. Framing (gain = 0, loss = 1) and first fixation (sure = 0, gamble = 1) were dummy-coded. Participants were treated as a random effect with varying intercepts ($s^2 = 1.21$, SD = 1.10) and varying slopes for frame ($s^2 = 0.17$, SD = 0.41) and first fixation ($s^2 = 1.17$, SD = 1.08). A random slope for expected value did not improve model fit.

Parameter	Estimate	SE	*z*	*p*
Intercept	-0.62	0.19	-3.35	< .001
Expected Value	-1.93	0.46	-4.18	< .001
Frame	0.90	0.13	7.19	< .001
First Fixation	0.85	0.20	4.20	< .001
Expected Value × Frame	2.13	0.73	2.93	.003
Expected Value × First Fixation	-0.54	0.74	-0.72	.47
Frame × First Fixation	-1.43	0.15	-9.44	< .001
Expected Value × Frame × First Fixation	-3.97	1.05	-3.78	< .001

Linkages between Analyses and Preregistration

In our online preregistration for this research project (https://osf.io/7j6kh/), we outlined a number of primary, tentative, and exploratory hypotheses. To facilitate comparison between our preregistration and the specific analyses included in this report, we provide a table mapping our reported analyses to preregistered hypothesis. To meet journal space constraints and for the sake of clarity and concision, we focused our analyses in this manuscript on what we viewed to be most central to our preregistered *core theoretical prediction* (see preregistration).

Table S7. Mappings between Reported Analyses and Preregistered Hypothesis

Preregistered Hypothesis	Analysis	Hypothesis Supported?	Notes
P-H1	2 (frame: gain, loss) × 2 (time constraint: none, 1s) repeated-measures ANOVA on the proportion of choices where the gamble was selected	Yes	A main effect of frame in the ANOVA was the critical test for P-H1.
P-H2	2 (frame: gain, loss) × 2 (time constraint: none, 1s) repeated-measures ANOVA on the proportion of choices where the gamble was selected	Yes	A frame × time constraint interaction with a larger difference between frames under TC was the critical test.
P-H3	2 (frame) × 2 (time constraint) repeated-measures ANOVA on the proportion of trials where the gamble was fixated first	Yes*	*The increase in first fixations to the sure option under TC only occurred on gain-framed trials.
P-H4	Mixed-effects logistic regression predicting choosing the gamble with frame, time constraints, first fixation, and their interactions	Partially	We initially predicted that fixating the sure option first under TC would produce more framing-effect-consistent choices (cf., Kwak & Huettel, 2018). This was true for gain-framed, but not loss-framed trials. This result led to our post-hoc hypothesis that deciders are more likely to select their first fixated option under TC and that first fixation is guided by peripheral cues (see T-H3g).
T-H3g	Mixed-effects logistic regression predicting first fixating the gamble with frame × time constraint, probability × time constraint, and their simple effects	Partially	Rather than TC having weak effects on first fixation when gamble probabilities were extreme (i.e., close to 0 or 1 where the gamble is a solid color), the effect of TC on first fixations was strongest when the gamble probability made it more distinct from the current sure option.

References

Diederich, A., Wyszynski, M., & Traub, S. (2020). Need, frames, and time constraints in risky decision-making. *Theory and Decision*. https://doi.org/10.1007/s11238-020-09744-6

Guo, L., Trueblood, J. S., & Diederich, A. (2017). Thinking fast increases framing effects in risky decision making. *Psychological Science*, *28*(4), 530–543. https://doi.org/10.1177/0956797616689092

Kwak, Y., & Huettel, S. (2018). The order of information processing alters economic gain-loss framing effects. *Acta Psychologica*, *182*, 46–54. https://doi.org/10.1016/j.actpsy.2017.11.013
